# Triple Network Model Dynamically Revisited: Lower Salience Network State Switching in Pre-psychosis

**DOI:** 10.3389/fphys.2020.00066

**Published:** 2020-02-11

**Authors:** Thomas A. W. Bolton, Diana Wotruba, Roman Buechler, Anastasia Theodoridou, Lars Michels, Spyros Kollias, Wulf Rössler, Karsten Heekeren, Dimitri Van De Ville

**Affiliations:** ^1^Institute of Bioengineering, École Polytechique Fédérale de Lausanne, Lausanne, Switzerland; ^2^Department of Radiology and Medical Informatics, Université de Genève, Geneva, Switzerland; ^3^Collegium Helveticum, ETH Zürich, Zurich, Switzerland; ^4^The Zürich Program for Sustainable Development of Mental Health Services, Psychiatry University Hospital Zürich, Zurich, Switzerland; ^5^Department of Neuroradiology, University Hospital Zürich, Zurich, Switzerland; ^6^Department of Psychiatry, Psychotherapy and Psychosomatics, University of Zürich, Zurich, Switzerland; ^7^Institute of Psychiatry, University of São Paulo, São Paulo, Brazil

**Keywords:** pre-psychotic, co-activation patterns, functional magnetic resonance imaging—fMRI, dynamic functional connectivity, default mode network (DMN), central executive network (CEN), salience network

## Abstract

Emerging evidence has attributed altered network coordination between the default mode, central executive, and salience networks (DMN/CEN/SAL) to disturbances seen in schizophrenia, but little is known for at-risk psychosis stages. Moreover, pinpointing impairments in specific network-to-network interactions, although essential to resolve possibly distinct harbingers of conversion to clinically diagnosed schizophrenia, remains particularly challenging. We addressed this by a dynamic approach to functional connectivity, where right anterior insula brain interactions were examined through co-activation pattern (CAP) analysis. We utilized resting-state fMRI in 19 subjects suffering from subthreshold delusions and hallucinations (UHR), 28 at-risk for psychosis with basic symptoms describing only self-experienced subclinical disturbances (BS), and 29 healthy controls (CTR) matched for age, gender, handedness, and intelligence. We extracted the most recurring CAPs, compared their relative occurrence and average dwell time to probe their temporal expression, and quantified occurrence balance to assess the putative loss of competing relationships. Our findings substantiate the pivotal role of the right anterior insula in governing CEN-to-DMN transitions, which appear dysfunctional prior to the onset of psychosis, especially when first attenuated psychotic symptoms occur. In UHR subjects, it is longer active in concert with the DMN and there is a loss of competition between a SAL/DMN state, and a state with insula/CEN activation paralleled by DMN deactivation. These features suggest that abnormal network switching disrupts one's capacity to distinguish between the internal world and external environment, which is accompanied by inflexibility and an excessive awareness to internal processes reflected by prolonged expression of the right anterior insula-default mode co-activation pattern.

## 1. Introduction

A fundamental feature of the healthy human brain is its intrinsic organization into coupled functional networks (Fox et al., 2005), which crucially includes coordinated default mode network (DMN) and central executive network (CEN) activity for cognitive and executive functions (Bressler and Kelso, 2001; Buckner et al., 2008). Their antagonistic activity putatively reflects competing modes of information processing (Fox et al., 2005; Fransson, 2005). The DMN, with its posterior cingulate cortex and medial prefrontal cortex hubs, serves untargeted inner thought (Andrews-Hanna, 2012); the CEN, particularly within the dorsolateral prefrontal and posterior parietal cortices, contributes to focused stimulus-dependent attention (Fox et al., 2005; Seeley et al., 2007; Menon and Uddin, 2010). The right anterior insula, the main salience network (SAL) driver, causally regulates this competing inter-network activity and thus facilitates both bottom-up perception and reorienting of attention, contributing as such to appropriate behavioral responses to salient stimuli (Menon and Uddin, 2010).

Disturbed DMN/CEN/SAL coordination has been related to the confusion of internally and externally focused attention and to the disturbance of cognition, as seen in psychotic disorders (Carhart-Harris and Friston, 2010; Nekovarova et al., 2014). In support of this, emerging evidence has attributed cognitive deficits in schizophrenia to dysfunctions in proper DMN/CEN coordination (Whitfield-Gabrieli et al., 2009; Chai et al., 2011; Whitfield-Gabrieli and Ford, 2012), whereas SAL anomalies have been mostly linked to reality distortion (Palaniyappan and Liddle, 2012; Pu et al., 2012), and thus posited to play a cardinal role in the development of psychotic symptoms (Palaniyappan et al., 2012a,b). Along the same line, we have recently demonstrated a loss of CEN-DMN anticorrelation, accompanied by DMN, CEN, and SAL anatomical overlap, in spontaneous brain activity of subjects at-risk for psychosis (Wotruba et al., 2014b). Importantly, those measures were associated differentially with cognitive (DMN/CEN) and psychopathological (SAL) symptoms.

A better understanding of the neurobiological underpinnings of different at-risk criteria is key to resolve possibly distinct harbingers of conversion to clinically diagnosed schizophrenia, and thus enable early intervention and lead to improved patient outcomes (Satterthwaite and Baker, 2015). Thus, different at-risk criteria have been conceptualized, including basic (BS) or ultra-high risk (UHR) symptoms. The former have been suggested to describe subtle, subclinical disturbances in mental processes, and to be the most direct self-experienced expression of the underlying neurobiological aberrations of schizophrenia; the latter, including subthreshold delusions and hallucinations, were conceptualized as secondary phenomena occurring at a later stage (Schultze-Lutter et al., 2016).

So far, few neuroimaging studies explore the relevance of these different conceptualizations, although divergent structural (Hurlemann et al., 2008; Koutsouleris et al., 2009a,b; Harrisberger et al., 2016) and functional (Ebisch et al., 2013, 2014; Wotruba et al., 2014a,b) brain abnormalities could be associated with different at-risk criteria.

Most resting-state studies in schizophrenia hypothesize stationary connectivity between brain regions, an oversimplification: for example, although the DMN and CEN are anticorrelated most of the time, they also exhibit some temporal intervals of correlated activity (Chang and Glover, 2010). Complex facets of brain activity, such as its non-stationary (de Pasquale et al., 2018; O'Neill et al., 2018) or scale-free (Van Den Heuvel et al., 2008; Van De Ville et al., 2010) nature, should thus be taken into account.

The growing field of network physiology—see Ivanov and Bartsch (2014) and Ivanov et al. (2016) for reviews—precisely attempts to quantify these complex properties. In the context of electroencephalography, approaches such as time-delay stability (Bashan et al., 2012; Bartsch et al., 2015) or delay-correction landscape (Lin et al., 2016) analyses characterize the nature and stability of the interactions between different network nodes, which jointly consist in different frequency rhythms at which functional processing occurs, and in organs under neural regulation (e.g., the eyes or the heart).

This sophisticated dynamics of brain function is modulated by the physiological state, as seen in the case of sleep stages (Bashan et al., 2012) or concomitant physical exercise and cognitive processing (Ciria et al., 2019). In addition, alterations are also seen in disease (Goldberger et al., 2002), which positions the study of resting-state functional brain dynamics as a question of particular interest regarding the emergence of pre-psychotic symptoms.

Recent electroencephalography work has shown the merits of probing the non-linear relationships between not just frequency rhythms, but also spatially remote brain centers (Liu et al., 2015). Functional magnetic resonance imaging (fMRI) is a particularly fitting imaging modality for this purpose, owing to its finer spatial resolution. Dedicated analytical strategies must be established, and accordingly, dynamic functional connectivity (Hutchison et al., 2013; Preti et al., 2017) analyses of schizophrenia (Damaraju et al., 2014; Du et al., 2016; Miller et al., 2016), and time-resolved fMRI investigations (Karahanoğlu and Van De Ville, 2017), have been gaining popularity.

However, the consideration of impairments at the level of specific network-to-network interactions, and so, possible maladaptive dynamics of the particular DMN/CEN/SAL relationship, remains subject to debate. Further, the analysis of connectivity, a second-order statistic, lowers the effective temporal resolution of the data, justifying recent developments toward frame-wise techniques to extract brain networks and analyse their interplays (Caballero-Gaudes et al., 2013; Karahanoğlu and Van De Ville, 2015).

Co-activation pattern (CAP) analysis (Liu and Duyn, 2013) is one such approach, where the sets of regions co-activating with a seed region of interest at different time points can be disentangled. To probe network-specific dynamic impairments, spatial or temporal features of the extracted CAPs can then be analyzed (Amico et al., 2014; Chen et al., 2015).

To improve the early recognition of psychosis and clarify the underlying role of the right anterior insula and co-activating regions, we carried resting-state fMRI on 25 subjects at-risk for psychosis with BS criteria, with full insight into their abnormal nature (Klosterkötter et al., 2001; Schultze-Lutter et al., 2016); 18 individuals with attenuated and/or brief intermittent psychotic symptoms (Yung and Mcgorry, 2007)—UHR criteria; and 29 healthy controls (CTR), matched on premorbid intelligence, age, handedness, and gender. We extracted six recurring CAPs, and given our specific interest in the interplay between the SAL, DMN, and CEN, focused the analysis on related ones, comparing their temporal expression across groups. We considered normalized counts, informing on the relative occurrence of each CAP, and duration, characterizing the average time for which a CAP is sustained. To assess the putative loss of competing relationship between CEN/DMN/SAL-containing CAPs in the at-risk stages for psychosis, we also quantified the balance in occurrence between these patterns of interest.

## 2. Materials and Methods

### 2.1. Participants

The present study included 76 participants (29 CTR, 28 BS, 19 UHR), and was approved by the local ethics committee of Zürich. Risk groups were recruited in the Swiss region of Zürich within the context of a larger study on early psychosis recognition (Theodoridou et al., 2014). Following an initial screening, in-person diagnostic interviews were administered, and a complete project description was provided. All subjects provided their written, informed consent. The procedure and imaging data acquisition were identical to those used in a previous study (Wotruba et al., 2014b).

Participants reporting at least one cognitive-perceptive basic symptom or at least two cognitive disturbances, as assessed by the Schizophrenia Proneness Interview (Schultze-Lutter et al., 2007) or, for adolescents (age < 18), by the Schizophrenia Proneness Interview “Child and Youth” version (Fux et al., 2013), fulfilled the inclusion criteria for the BS status. Those describing at least one attenuated psychotic symptom or brief, limited, intermittent psychotic symptoms, as assessed by the Structured Interview for Prodromal Syndromes (Miller et al., 2003), fulfilled the criterion for UHR status. Five BS and four UHR subjects were taking second-generation (atypical) antipsychotic medication at the time of scanning. Chlorpromazine equivalents were calculated for them (Andreasen et al., 2010). Five BS and three UHR subjects were being treated with an antidepressant. Our healthy CTRs were screened with the Mini-International Neuropsychiatric Interview (Sheehan et al., 1998) to ensure that none had any current or prior history of psychiatric illness. Those receiving any medication were excluded.

Exclusion criteria were contraindications against MRI, pregnancy, a history of neurological illness, as well as drug or alcohol dependence. Structural MRI scans were checked by an experienced neuroradiologist, excluding participants with structural brain abnormalities. The groups had a mean estimated premorbid intelligence slightly above average, as assessed using a German test for estimating premorbid, verbal intelligence—Mehrfachwortschatz Test, Version B (Lehrl, 1999); adolescent subjects (age < 20) adopted the Leistungsprüfsystem-3 German test for fluid, nonverbal intelligence (Horn, 1983). Handedness was examined by the Edinburgh Handedness questionnaire (Oldfield, 1971).

A list of all 76 subjects, including their taken antipsychotic drugs/antidepressants and exclusion criteria—as further detailed below—is provided in Table S1.

### 2.2. Imaging Data Acquisition

Resting-state fMRI data were acquired at the University Hospital of Psychiatry Zürich, Switzerland, using a Philips Achieva TX 3 T whole-body MR unit with an eight-channel head coil. Functional scans (6 min runs) involved a sensitivity-encoded single-shot echo-planar (factor 1) $T_{2}^{*}$-weighted echo-planar imaging sequence [repetition time = 2,000 ms; echo time = 35 ms; field of view = 220 × 220 mm^2^; acquisition matrix = 88 × 87, interpolated to 128 × 128; 32 contiguous slices with a spatial resolution of 2.5 × 2.5 × 4 mm^3^ (reconstructed 1.72 × 1.72 × 4 mm^3^); flip angle θ = 78°; and sensitivity-encoded acceleration factor *R* = 1.8]. We also acquired three-dimensional *T*_1_-weighted anatomical images [160 slices; TR = 1,900 ms; TE = 2.2 ms; inversion time = 900 ms; θ = 78°; spatial resolution of 1 × 1 × 1 mm^3^ (reconstructed 0.94 × 0.94 × 1 mm^3^); field of view = 240 × 240 mm^2^]. Echo-planar imaging sequences were conducted under darkness; participants were asked to keep their eyes closed, lying quietly without falling asleep, as confirmed after scanning. To minimize arousal and anxiety effects, acquisition started 10 min after moving the subjects to their final MR bore positioning.

### 2.3. Preprocessing

Preprocessing was conducted using SPM8 (Wellcome Department of Imaging Neuroscience, London, UK; http://www.fil.ion.ucl.ac.uk/spm), running in Matlab (Mathworks Inc., Sherbon, MA, USA), and the conn toolbox pipeline (version 15a). We performed realignment, slice timing correction, co-registration to structural *T*_1_ scan, spatial normalization to Montreal Neurological Institute space, and spatial smoothing (6 mm full width at half maximum). Spurious sources of noise were estimated by the anatomical component-based noise reduction strategy (Behzadi et al., 2007), and included with the six movement parameters as first level nuisance co-variates in a General Linear Model. Temporal band-pass filtering (0.01–0.10 Hz) and z-scoring were applied, and framewise displacement was quantified using Power's criterion (Power et al., 2012). We excluded three subjects (2 BS and 1 UHR) for whom more than 20% of volumes were censored.

### 2.4. Co-activation Pattern Analysis

#### 2.4.1. Seed Choice, Frame Selection, and Extraction of Co-activation Patterns

**Figure 1:**
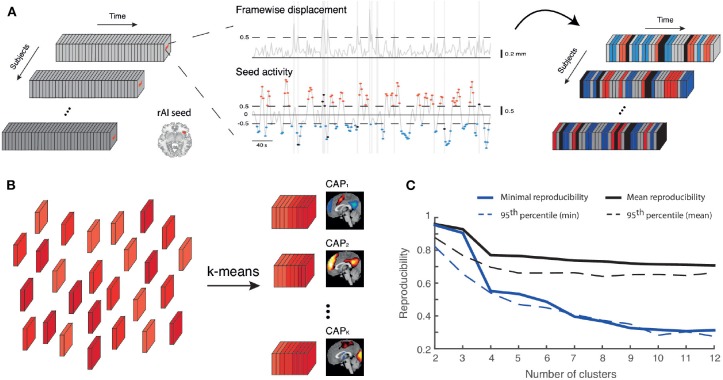
Co-activation pattern analysis. **(A)** A right anterior insula (rAI) activity time course (middle panel, bottom trace) is computed for each subject, and the frames for which it exceeds a threshold *T* = 0.5 positively or negatively (respectively red or blue) are tagged. Frames corrupted by a framewise displacement (middle panel, top trace) larger than 0.5 mm are also tagged in black. Here, only non-corrupted activation frames are analyzed. **(B)** Retained frames across subjects (depicted by different shades of red) undergo k-means clustering to be separated into *K* different co-activation patterns (CAPs), each the arithmetic mean between a subset of frames denoting one particular set of regions with which the seed was strongly co-active at some time points. **(C)** Mean (black) and minimal (blue) reproducibility for cluster number ranging from 2 to 12, computed as the average over 100 separate trials (plain lines), and for the same measures, 95^th^ percentiles of the related null distributions (dashed lines). The selected value of *K* = 6 shows mean and minimal reproducibility values above this null data threshold.

Figure 1 illustrates the key methodological steps underlying CAP analysis. Based on previous analyses (Wotruba et al., 2014b) and recently proposed theoretical models for disturbance in the triple network (Menon, 2011), we selected a right anterior insula seed. A spherical region (8 mm radius, Montreal Neurological Institute coordinates: x = 38, y = 22, z = −10) based on previous studies (Seeley et al., 2007; Woodward et al., 2011) was created with the MARSBAR toolbox (http://marsbar.sourceforge.net/), and had its activity thresholded to solely retain frames with signal larger than *T* = 0.5 (seed activation). To rule out biases due to a different number of selected frames across groups, we also computed the amount of baseline, unselected time points, and included it as a co-variate in our analyses.

K-means clustering was applied to the retained frames. If *C*(*i, j*) is Pearson's spatial correlation coefficient between fMRI volumes *i* and *j*, then *d*(*i, j*) = 1 − *C*(*i, j*) was used as a distance measure. The algorithm was run 20 separate times, to avoid being trapped in a local optimum. Each run was performed with a maximal limit of 100 iterations, using randomly selected data points for initialization. Similarly to previous CAP studies (Liu and Duyn, 2013; Liu et al., 2013), only the 15% most active and 5% most deactive voxels in each fMRI volume were considered for clustering, in order to damp the impact of noisy dimensions on the clustering process, and all remaining non-null clusters with <6 neighboring elements were also discarded to further attenuate noise.

Through k-means clustering, each of the retained frames is assigned to an output cluster from the algorithm. Denoting a vectorized fMRI volume *i* by **F**_*i*_, the set of frames assigned to cluster *k* by $K_{k}$, and the number of frames assigned to cluster *k* by *N*_*k*_, a CAP is defined as the arithmetic mean between all the frames attributed to the same cluster:

(1) $$\begin{array}{l} {\text{CAP}}_{k}=\frac{\sum_{i\in K_{k}}{\text{F}}_{i}}{N_{k}}. \end{array}$$

**Figure 2:**
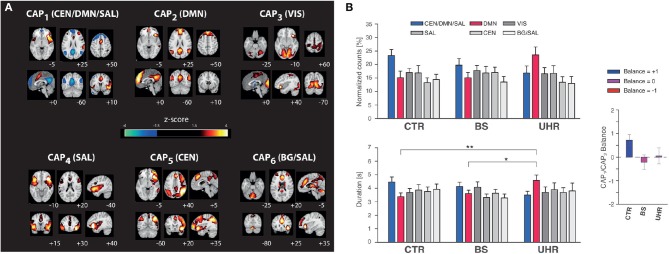
Altered co-activation pattern temporal features in subjects at risk for psychosis. **(A)** The six co-activation patterns (CAPs) found for a right anterior insula seed, with Montreal Neurological Institute slice coordinates (bottom right of each slice). Left on the figure stands for the left side of the brain. CEN, central executive network; DMN, default mode network; SAL, salience network; VIS, visual network; BG, basal ganglia. **(B)** Normalized counts (top left graph), duration (bottom left graph), and balance (right graph) metrics for all six co-activation patterns in healthy controls (CTR), subjects with basic symptoms of psychosis (BS), and subjects at ultra-high risk for psychosis (UHR). Normalized counts for CAP_1_ (*p* = 0.06) and CAP_2_ (*p* = 0.02) trended toward significance for a group difference. Duration trended toward significance for CAP_1_ (*p* = 0.06), and was significant for CAP_2_ (*p* = 0.002), with *post-hoc* tests revealing CTR vs. UHR (*p* = 0.001) and BS vs. UHR (*p* = 0.02) differences. Balance was significantly different across groups (*p* < 0.05). Error bars indicate standard error of the means. *p < 0.05, **p < 0.005.

Finally, in order to be able to delineate spatial territories showing significant seed co-(de)activation, each CAP map was individually subjected to spatial z-scoring. A threshold of 1.5 was chosen, in accordance with previous work (Karahanoğlu and Van De Ville, 2015), and also used for visualization (see Figure 2A).

#### 2.4.2. Determination of the Number of Clusters

To estimate an optimal cluster number *K*, we randomly split our subject population into two equally-sized groups 100 times; each time, clustering was performed separately on each half, for *K* = 2–12. Best-match pairs between each group's CAPs were established (Kuhn, 1955), and lowest/mean correlation values (averaged over the 100 trials) were considered as two reproducibility measures.

To generate reproducibility null distributions, we created 1,000 sets of surrogate data under a stationarity assumption: white noise with the same variance as the real data was added to a subject-specific stationary activity pattern estimated from the first eigenvector of its spatial covariance matrix, and randomly scaled over time. Following temporal z-scoring of this surrogate data, null distributions for both assessed reproducibility measures were generated as described above.

#### 2.4.3. Metrics to Quantify CAP Dynamics

For each CAP, we computed two dynamically informative subject-specific metrics: (1) normalized count, equal to 0/1 if the considered CAP never occurs/occurs every time the right anterior insula seed is active; (2) duration [s] (average time during which the CAP remains active). To assess a possible CAP_1_/CAP_2_ competing relationship, a balance metric was also computed for each subject as log_2_(CAP_1_ counts/CAP_2_ counts). Positive/negative values reflect a dominance of CAP_1_/CAP_2_.

### 2.5. Statistical Analyses

A one-way multivariate analysis of variance (MANOVA) was conducted, with and without baseline counts/chlorpromazine equivalents as co-variates, to determine group differences in CAP_1_ and CAP_2_ normalized counts and duration. We identified one BS univariate outlier, as assessed by inspection of a box plot for values >3 box-lengths from the edge. This subject, who reported intake of relaxane prior to scanning, was taken out of the analysis, because MANOVA is sensitive to the effect of outliers. This resulted in a total of 72 subjects (following scrubbing-related exclusion) matched across groups for handedness, gender, age, and IQ (Table 1).

**Table 1:** Demographic characteristics and rating of symptoms for the analyzed subjects.

	**CTR**	**BS**	**UHR**	**Statistical evaluation**
*N*	29	25	18	
Gender (f:m)	13:16	8:17	9:9	χ^2^ = 1.6, *n*.*s*.
Handedness (r:l:b)	25:2:2	22:1:2	17:1:0	χ^2^ = 1.7, *n*.*s*.
Age (years)	22.8 ± 5.0	21.0 ± 4.5	20.8 ± 4.1	*F* = 1.6, *n*.*s*.
Estimated intelligence	111.7 ± 14.5	101.0 ± 11.6	95.0 ± 7.1	*F* = 0.9, *n*.*s*.
SIPS (Positive)	–	4.7 ± 2.8	9.0 ± 4.3	*U* = −3.4, *p* < 0.001
SIPS (Negative)	–	7.8 ± 4.6	11.9 ± 5.7	*U* = −2.4, *p* = 0.1
SIPS (General)	–	6.2 ± 2.8	7.5 ± 3.9	*U* = −1.5, *n*.*s*.
SIPS (Disorganization)	–	2.8 ± 2.1	4.4 ± 2.0	*U* = −2.9, *p* = 0.004
GAF	–	64.7 ± 12.9	59.8 ± 11.9	*U* = −1.1, *n*.*s*.
CPZ equivalents	–	18.00 ± 51.5	118.3 ± 490	*T* = 1.2, *n*.*s*.

*The 29 healthy controls (CTR), 25 subjects with basic symptoms of psychosis (BS), and 18 subjects at ultra-high risk for psychosis (UHR) considered in this study are matched for gender balance and handedness, as assessed by Pearson's χ^2^ test. They are also matched for age and estimated intelligence, as determined with a three-level analysis of variance test. The extent of symptoms according to the Structured Interview for Prodromal Syndromes (SIPS), and the Global Assessment of Functioning (GAF) scale mean, are also provided for BS and UHR subjects, who are compared by a Mann–Whitney U-test. Finally, chlorpromazine (CPZ) equivalents are also shown, contrasting both groups with a t-test. Data is presented as mean ± standard deviation where appropriate*.

Baseline, balance, duration, and counts data were normally distributed (Shapiro-Wilk test, *p* > 0.05), except for CTR CAP_1_ duration (*p* = 0.01) and CAP_2_ counts (*p* = 0.02), both positively skewed. This was tolerated, as MANOVA is considered robust to non-normality, with only a small effect on Type I error (Weinfurt, 1995). MANOVA analysis was judged appropriate, as there were no multivariate outliers seen by box plot and Mahalanobis distance (*p* > 0.001), no multi-collinearity (*r* < 0.65), and homogeneity of variance-covariance matrices (Box's M test, *p* > 0.001). Linear relationships were always found as assessed by scatter plot, except for BS CAP_1_ duration and counts, which may have resulted in loss of power.

The CAP metrics differing significantly between groups were used to determine the relation to clinical symptom sub-scores, as assessed by the Schizophrenia Proneness Interview and Structured Interview for Prodromal Syndromes. We avoid reporting any significance values and thus take into account the issue of circular analysis (Kriegeskorte et al., 2009), as the BS and UHR groups were selected based on such criteria.

Data is presented as mean ± standard error of the mean, unless otherwise stated.

## 3. Results

### 3.1. Right Anterior Insula Co-activation Patterns

The right anterior insula was subjected to CAP analysis with *K* = 6 (see Figure 1C) to identify co-activating regions. 20.1% of retained frames were attributed to CAP_1_, 17.8% to CAP_2_, 17.0% to CAP_3_, 16.9% to CAP_4_, 14.7% to CAP_5_, and 12.5% to CAP_6_. CAP_1_ displayed typical CEN (right dorsolateral prefrontal cortex, posterior parietal cortex) and SAL hubs, as well as negative DMN signal. In contrast, CAP_2_ predominantly displayed positive signal from DMN areas. CAP_3_ included a visual network, CAP_4_ featured SAL areas, CAP_5_ contained CEN regions, and CAP_6_ was a mix between SAL and basal ganglia nodes. Due to their frequent occurrence and specific link to the CEN/DMN/SAL relationship, CAP_1_ and CAP_2_ dynamic features were further analyzed.

### 3.2. Quantification of CAP Dynamics

With one-way MANOVA, we found statistically significant group differences on the combined dependent variables (*F* = 2.2, *p* = 0.02; Pillai-trace = 2.4; partial η^2^ = 0.13). Follow-up univariate ANOVAs showed that CAP_2_ duration (*F* = 7.2, *p* = 0.002, partial η^2^ = 0.18) differed significantly between groups (Bonferroni-adjusted α-level 0.0125), while all other variables trended toward significance (CAP_1_ duration: *F* = 2.9, *p* = 0.06, partial η^2^ = 0.08; CAP_1_ counts: *F* = 2.9, *p* = 0.06, partial η^2^ = 0.08; CAP_2_ counts: *F* = 4.1, *p* = 0.02, partial η^2^ = 0.11).

After adjustment for baseline counts and chlorpromazine equivalents, we similarly found CAP_2_ duration significance (*F* = 6.7, *p* = 0.002, partial η^2^ = 0.18), while other variables trended toward significance (CAP_1_ duration: *F* = 2.8, *p* = 0.07, partial η^2^ = 0.08; CAP_1_ counts: *F* = 3.2, *p* = 0.04, partial η^2^ = 0.09; CAP_2_ counts: *F* = 4.1, *p* < 0.02, partial η^2^ = 0.12).

Tukey-Kramer *post-hoc* tests revealed a significant difference in CAP_2_ duration between CTR (3.4 ± 1.2 s) and UHR (4.6 ± 0.3 s) subjects (*p* = 0.001), and between BS (3.6 ± 0.2 s) and UHR individuals (*p* = 0.02), but no difference between CTR and BS groups (*p* = 0.4; see Figure 2B, bottom left graph).

### 3.3. Balance Between CAP_1_ and CAP_2_

The CAP_1_/CAP_2_ balance metric was positive for CTR (0.72 ± 0.23), negative for BS (−0.22 ± 0.3), and close to zero for UHR subjects (0.05 ± 0.34; see Figure 2B, right graph). A one-way ANOVA revealed statistically significant group differences (*F* = 3.3, *p* < 0.05, partial η^2^ = 0.09), which remained after adjusting for chlorpromazine equivalents (*F* = 3.4, *p* < 0.05, partial η^2^ = 0.09).

### 3.4. Association of CAP Metrics to Clinical Symptom Scores

CAP_2_ duration was related to the severity of psychopathological symptoms in at-risk subjects. For the Structured Interview for Prodromal Syndromes ratings, we found associations to disorganization symptoms (ρ = 0.3) such as odd behavior or trouble with focus, and to positive symptoms (ρ = 0.3) such as unusual thought content, persecutory delusions, perceptional abnormalities, or disorganized communication. We also found associations with the Schizophrenia Proneness Interview sub-score for disturbances in experiencing the self and surroundings (ρ = 0.3), including decreased emotional discrimination abilities or increased emotional reactivity in response to routine social interactions.

## 4. Discussion

An aberrant orchestration within the triple network (DMN/CEN/SAL) has been suggested as a backbone for features of various psychiatric disorders (Menon, 2011). Viewing the brain as a dynamic system flexibly adapting to changes in internal and external states (Dixon et al., 2016), we analyzed the dynamic interplay of right anterior insula-driven networks, characterizing and quantifying these states in subjects reporting different at-risk for psychosis criteria.

Our analysis revealed that across intrinsic states, the insular seed activates over the entire resting-state scan, across subjects, in concert with well-known networks: in agreement with prior studies (Nomi et al., 2016), CAP_3_ and CAP_6_ displayed visual and basal ganglia networks. Thus, the right anterior insula may be working together with those to coordinate sensory information, with CAP_6_ supporting the view that dopaminergic paths are involved in insular activity (Menon and Uddin, 2010; Shine et al., 2013). Most frequently, we found that our insular seed fluctuated between a state of antagonistic relationship to the DMN in CAP_1_, which also included SAL-TPN co-activation, and a state of co-activation with the DMN in CAP_2_. This is in line with recent dynamic studies (Karahanoğlu and Van De Ville, 2015; Nomi et al., 2016), but contradicts the findings of Ryali et al. (2016), who showed segregation of the SAL from a DMN-CEN state, possibly owing to a different computational approach. In any case, we confirm the pivotal role of the SAL to guarantee the balance between the DMN and CEN by integrating information (Menon, 2011; Nekovarova et al., 2014; Uddin, 2015), as hypothesized by the triple network model, which implies a fundamental neural constraint on cognition, assuming that this constitution of antagonistic network relationships reflects the competition between external and internal information processing (Smallwood et al., 2013).

Due to our specific interest in the networks involving the CEN/DMN/SAL, we focused on the related co-activation patterns, CAP_1_ and CAP_2_. UHR subjects exhibited longer durations of excursions in the CAP_2_ state (right anterior insula/DMN co-activation), and also, with a trend toward significance, larger counts. This is somewhat in line with a report on young subjects with sub-threshold psychosis-spectrum symptoms (Satterthwaite and Baker, 2015), who showed diminished connectivity within the SAL, but enhanced connectivity within the DMN. The right anterior insula has been ascribed a central role for bottom-up processing through interoceptive paths, possibly through the basal ganglia, assisting target brain regions in generating appropriate behavioral responses to salient stimuli via the CEN (Menon and Uddin, 2010), while the DMN is a key structure for awareness and source attribution (Northoff et al., 2006; Leech and Sharp, 2013), and reflects internally guided, self-referential thoughts (Zabelina and Andrews-Hanna, 2016). Thus, the overly potent role of the right anterior insula in engaging with the DMN in UHR may reflect an increasing shift to the internal focus of attention. According to the proximal salience hypothesis, as well as the source monitoring model (Menon, 2011; Damaraju et al., 2014; Robinson et al., 2016), this process might lead to the externalization of thoughts at a later stage of symptom progress and distort the attribution of agency, explaining a number of the positive symptoms of schizophrenia.

The significant group difference seen between BS and UHR in terms of CAP_2_ duration may relate to the neurodevelopmental etiology of psychosis, as basic symptoms are presumed to characterize the early, and UHR, the late, prodromal phase (Fusar-Poli et al., 2013). Thus, this finding could account for the symptomatic worsening and cognitive decline on the path to a psychotic outbreak, as paralleled by the finding of UHR samples exhibiting more frequent and more pronounced neurocognitive impairments compared to samples with BS symptoms (Schultze-Lutter et al., 2016). Moreover, this network feature then reflects an increased risk toward a psychotic outbreak, rather than a trait or an endophenotype.

Another important difference to draw for those two at-risk groups is further substantiated by the analysis of the balance metric. Here, we show that in contrast to the controls and BS, the UHR do not exhibit a competitive relationship between CAP_1_ and CAP_2_. Similarly, children also revealed longer persistence in individual SAL, CEN, and DMN states, and reduced state switching probability (Ryali et al., 2016), pointing to an immature brain network organization in UHR (Satterthwaite and Baker, 2015), perhaps reflecting a state of inflexibility to adapt in a constantly changing environment (Dajani and Uddin, 2015). The loss of CAP_1_ dominance may be caused by weakened top-down regulation by CEN regions, as supported by the observation of failure of reciprocal influence between the right anterior insula and the dorsolateral prefrontal cortex in schizophrenia (Palaniyappan et al., 2013). Additionally, a takeover of the CEN in favor of voluntary process in a destabilized triple network in hallucinating patients was followed by discontinuation of hallucinations (Lefebvre et al., 2016). Albeit speculative, an initial loss in dominance of CAP_1_ (frontal and salience-related regions under active inhibition of the DMN) in the early at-risk stage (BS) might be followed by further disintegration of the triple network, as ego disturbances and other related psychotic symptoms (Lebedev et al., 2015) become more advanced in UHR. Future longitudinal studies leveraging the present analytical approach to a finer prospective cohort design will be important to confirm whether a failure in dynamic direct connection, as suggested, is accompanied by worsening of psychotic symptoms (Satterthwaite and Baker, 2015).

The loss of network competition could reflect the risk of a breakdown of separateness of one's internal world and external environment (Carhart-Harris et al., 2013; Nelson et al., 2014). Corroborating this, we found an association of CAP_2_ duration to positive (psychotic) and disorganized symptoms (such as odd appearance, bizarre thinking, unusual ideas, or attentional troubles), but also to sub-scores for disturbances in experiencing the self and surroundings.

Our findings remained when controlling for baseline right anterior insula activity, demonstrating that they involve the engagement of this seed within different network constellations, rather than mediation of insular activity *per se*. In addition, as some of the analyzed subjects were medicated and given the vivid debate on the extent to which antipsychotic medication contributes to changes in resting-state fMRI (Sambataro et al., 2010), we also controlled for chlorpromazine equivalents; all observed group differences were still present. While other medications (e.g., antidepressants) may also exert a modulatory influence on resting-state functional properties (McCabe and Mishor, 2011; Arnone et al., 2018), they were taken by only a minor fraction of the analyzed subjects (see Table S1), and thus, this factor also likely played a minimal role on our results.

In future work, a bigger sample will be necessary to learn more about the neurobiological underpinnings of different stages within the disease trajectory. In accordance with this, we did find a trend toward significant group differences in both CAP_1_ metrics, and also for CAP_2_ counts (applying a conservative Bonferroni-adjusted significance threshold). Thus, it is unclear whether a bigger sample size would also reveal BS-specific features, as nonlinear relationships for CAP_1_ duration and counts in this group may have resulted in loss of statistical power.

## 5. Conclusion

In summary, our findings substantiate the pivotal role of the right anterior insula in governing transitions between CEN and DMN, which appear dysfunctional prior to the onset of psychosis, especially when first attenuated psychotic symptoms occur. In the UHR stage, this is in at least two ways: firstly, the right anterior insula is longer active in concert with the DMN, and secondly, there is a loss of competition between a SAL/DMN state, and a state with insular/CEN activation paralleled by DMN deactivation, denoting decreased state switching. Considered jointly with our recent stationary analysis of the triple network (Wotruba et al., 2014b), these features suggest that abnormal network switching disrupts one's capacity to distinguish between the internal world and external environment, which is accompanied by inflexibility and an excessive awareness to internal processes (reflected by prolonged expression of the right anterior insula/DMN co-activation pattern). This may, in turn, contribute to the rise in psychotic perceptions. More generally, our work demonstrates the mechanistic relevance of dynamic transitions between temporal network states to support healthy cognition.

## Data Availability Statement

The datasets analyzed in this article are not publicly available. Requests to access the datasets should be directed to Thomas Bolton, thomas.bolton@epfl.ch.

## Ethics Statement

The studies involving human participants were reviewed and approved by the local ethics committee of Zürich. The patients/participants provided their written informed consent to participate in this study.

## Author Contributions

TB and DW jointly designed the research, performed the analyses, and wrote the manuscript. RB, AT, LM, SK, WR, and KH were involved in the follow-up of the patients and acquired the analyzed data. DV surveyed the work, contributed to the analyses, and interpretation of the results.

### Conflict of Interest

The authors declare that the research was conducted in the absence of any commercial or financial relationships that could be construed as a potential conflict of interest.
