# Supplementary material for: Triple Network Model Dynamically Revisited: Lower Salience Network State Switching in Pre-psychosis
Source: Front Physiol. 2020 Feb 11;11:66. doi: 10.3389/fphys.2020.00066 (PMC7027374; doi:10.3389/fphys.2020.00066)
Supplement: Supplementary file 1 [file Data_Sheet_1.PDF]

frontiers

Supplementary Material

SUPPLEMENTARY TABLES

Index	Group	Antipsychotic (Dose [mg])	Antidepressant (Dose [mg])	Exclusion criteria
E128	UHR		Sertralin (50), Trittico (150)	
E129	UHR			
E130	BS	Seroquel (100), Temesta (1)		
E131	UHR	Seroquel (300), Cipralex (10)		
E132	BS			
E133	BS			
E134	BS		Trittico (100)	
E136	BS			
E137	UHR		Fluoxetine (100)	
E140	BS			
E143	BS			
E144	BS			
E146	BS			
E147	UHR	Invega (3)		
E149	BS			
E150	BS			Relaxane intake
E151	BS	Seroquel XR (50), Cipralex (20)		
E154	BS	Seroquel (25)		
E155	UHR			
E156	BS		Zoloft (75), Deroxat (100)	
E157	BS	Seroquel (25)	Trittico (50)	Excessive motion
E159	UHR			
E161	UHR			
E163	BS		Zoloft (75)	
E165	BS	Seroquel XR (50)	Effexor (225)	
E166	UHR			
E167	UHR			
E170	BS			
E173	UHR			
E175	UHR	Risperdal (0.25)		
E176	BS			
E177	UHR			
E178	BS			
E179	BS			
E180	UHR	Abilify (10)		
E181	UHR			
E182	UHR			
E183	BS			
E184	BS			
E186	BS			Excessive motion
E189	BS			
E191	BS			
E192	BS			

E196	UHR		Fluctin (20)	
E197	UHR			
E198	UHR			Excessive motion
E199	BS			
K006B	CTR			
K015	CTR			
K017	CTR			
K020	CTR			
K021	CTR			
K022	CTR			
K024	CTR			
K027	CTR			
K029	CTR			
K030	CTR			
K033	CTR			
K034	CTR			
K036	CTR			
K037	CTR			
K038	CTR			
K042	CTR			
K043	CTR			
K044	CTR			
K045	CTR			
K047	CTR			
K048	CTR			
K049	CTR			
KJ001	CTR			
KJ002	CTR			
KJ005	CTR			
KJ010	CTR			
KJ016	CTR			
KJ017	CTR			
KJ018	CTR			

Table S1: List of all 76 subjects initially considered in this work, with details regarding the taken antipsychotic drugs and antidepressants, as well as exclusion criteria whenever applicable. Three subjects (2 BS, 1 UHR) were excluded because of excessive motion (more than 20% of frames scrubbed out), and one BS subject was excluded due to the intake of relaxane prior to scanning, resulting in outlier values for the assessed metrics. Other medications not listed in the table include Focalin (E154), Ritalin (E154, E156, E196), Temesta (E170), Strattera (E175), and Redormin (E180). CTR: healthy controls. BS: subjects with basic symptoms of psychosis. UHR: subjects at ultra-high risk for psychosis.
